# A Video Analysis of Suspected Injuries and Suspected Concussions in Elite Ladies Gaelic Football Matches

**DOI:** 10.1177/19417381251372982

**Published:** 2025-09-25

**Authors:** Leigh Porter, Stephen W. West, Stephen Behan, Siobhán O’Connor

**Affiliations:** † Centre for Injury Prevention and Performance, School of Health and Human Performance, Dublin City University, Dublin, Ireland; ‡ Centre for Health, and Injury and Illness Prevention in Sport, University of Bath, Bath, UK; § UK Collaborating Centre on Injury and Illness Prevention in Sport, University of Bath, Bath, UK; ‖ Sport Injury Prevention Research Centre, Faculty of Kinesiology, University of Calgary, Calgary, Canada; ¶ School of Health and Human Performance, Faculty of Science and Health, Dublin City University, Dublin, Ireland; # Insight Research Ireland Centre for Data Analytics, Dublin City University, Dublin, Ireland

**Keywords:** concussion, female Gaelic football, injury, video analysis

## Abstract

**Background:**

Previous research shows that injuries are prevalent in ladies Gaelic football. However, little is known about how these injuries occur (ie, the mechanism of injury). In addition, there are limited data on injuries sustained during elite-level matches. Concussions are also a key concern, yet research has examined solely self-reported suspected concussions, and it remains unclear how potential concussions are identified and managed during matches.

**Objective:**

To establish the incidence, characteristics, and management of suspected injuries and concussions in elite ladies Gaelic football matches.

**Design:**

Cross-sectional video analysis study.

**Level of Evidence:**

Level 3.

**Methods:**

A video coding framework was developed based on similar published studies and validated by 5 Gaelic football-specific raters. One research assistant coded all matches from the 2022 season, and an experienced referee also reviewed foul play events.

**Results:**

There were 829 suspected injuries (suspected injury rate [IR], 229.0 per 1000 hours; 95% CI, 214.0-245.2; 6.9 suspected injuries per match) and 162 suspected concussions (IR, 44.8 per 1000 hours; 95% CI, 38.4-52.2; 1.4 per match) recorded in 120 matches. Most suspected injuries received onfield medical attention (84.0%); however, just 13.6% of suspected concussions were removed from play. The tackle accounted for the most suspected injuries (40.2%), player-to-player contact (68.2%) was the most common mechanism, and the head/neck (38.1%) was the body location injured most frequently. Foul play concerned 53.2% of suspected injuries, with 76.7% of fouls concerning the tackle.

**Conclusion:**

The nonremoval of suspected concussions and the frequency of tackle-related suspected injuries and foul play warrants attention.

**Clinical Relevance:**

Developing and implementing injury prevention programs, concussion management strategies, and education for all knowledge users may contribute to a safer playing environment.

Ladies Gaelic football (LGF) is a popular amateur Irish field sport, ^46^ which, although played mainly in Ireland, has a growing strong presence internationally and is recognized as one of the fastest-growing sports in Europe.^27^ The Ladies Gaelic Football Association (LGFA) is the governing body for LGF.^37^ In adult players, the women’s elite (intercounty) competition involves amateur players who are selected from their local clubs to compete at the highest level for their respective county team in 2 major competitions; the National Football League and All-Ireland Championship.^26^ LGF is an invasion-based sport, similar to Australian football, where 2 15-player teams (1 goalkeeper, 6 backs, 2 midfielders, and 6 forwards) compete to score points (over the crossbar) or goals (under the crossbar) against the opposition over 2 30-minute halves.^24,32^ It is a fast-paced, multidirectional sport that requires players to run, jump, catch, turn, and tackle, as well as kick and hand-pass a round leather ball between their teammates. LGF is comparable with Gaelic football played by men but with some rule differences. For example, in LGF, players can pick up the ball from the ground using their hands, and deliberate bodily contact is prohibited.^24,32^ Given the demands of the game, there is an inherent risk of injury.^32^ Injuries in sport can negatively impact the physical and mental health of players,^33^ team performance, and success,^13^ and lead to financial costs,^37^ making them a significant burden for all knowledge users.

Most studies examining injury incidence have focused on male Gaelic footballers,^31,34,35,42^ with just 2 prospective studies completed on collegiate LGF players,^32,42^ another study examining LGFA injury fund data over 9 years,^37^ and 1 retrospective study conducted in the United States.^2^ Match injury incidence rates between 34.7 and 42.5 injuries per 1000 player hours for collegiate LGF have been reported, which is higher than rates of their male counterparts (25.1-28.5 match injuries per 1000 hours).^35,42^ Noncontact and lower limb injuries are most prominent in LGF.^32^ Although the breadth of research on injuries in LGF is growing, there are no prospective data on injury in matches at women’s elite level, and detailed information on the mechanism of injury and the inciting events is limited. Recent research has identified barriers and facilitators to injury prevention program uptake and implementation in LGF knowledge users^6^; however, to mitigate injury risk and develop effective injury prevention strategies for the LGFA, understanding how these injuries occur is a fundamental requirement.^15^

Concussion is a substantial issue across community sports, including LGF. A survey on elite and non-elite LGF players found that 52.5% of respondents reported previously sustaining at least 1 concussion while playing.^30^ Only 2 studies have examined the prevalence, characteristics, and management of concussions in LGF.^30,36^ In a study that examined self-reported concussions, across all levels of play, approximately one-sixth (17.5%) of players self-reported sustaining a suspected/diagnosed concussion during the previous season; however, elite players have an almost 2-fold greater likelihood of sustaining a self-reported concussion compared with nonelite players.^30^ The majority of concussions happen during matches,^30,32^ with contact with another player the mechanism self-reported most (62.9%).^30^ Onfield concussion management during matches is an essential component of the LGFA concussion guidelines and is particularly important at the elite level, where medical professionals are present and should be equipped to assess and remove players from play when appropriate. Worryingly, management of concussion appears to be poor, with research on intercounty men’s Gaelic football finding that typically only brief assessments take place onfield and players rarely are removed from play.^41^ Furthermore, in LGF, one-quarter of players with diagnosed concussions reported returning to play on the day of injury.^30^ Much of the research surrounding concussions in LGF are retrospective in design and rely on self-reporting, with no previous objective methods of identifying suspected concussions used to date.

Although prospective cohort research studies are optimal for longitudinal examination of injuries in sport, video surveillance has been used widely in various sports to understand injury characteristics such as their mechanisms and specific incidents.^1,5,11,16^ In women’s rugby union, video analysis has been validated and used to estimate the incidence of suspected injuries and suspected concussions, without any supporting prospectively collected injury data.^45^ Similarly, video analysis has been used to identify characteristics of potential concussive events in male intercounty Gaelic football and hurling.^39,40^ There is a clear need for injury data in LGF, so such methods could be effective in identifying the incidence and characteristics of suspected injuries in this sport. To date there is no prospective data on injuries in LGF matches at the elite level. Thus, this research aims to identify the rates of suspected injury and suspected concussion in intercounty LGF and establish the specific characteristics of injury and concussion and the onfield management of these injuries.

## Methods

### Study Design

A cross-sectional video analysis study on all intercounty championship and league LGF matches (*n* = 120) from the 2022 season was conducted. Ethical approval was granted by the Dublin City University Research Ethics Committee (DCUREC/2022/222).

### Procedures

A coding framework was developed based primarily upon a similar study in women’s rugby union,^45^ whereby the criteria for suspected injuries and suspected concussions was adopted. The mechanism of injury, event causing the injury, injury site, foul play, and whether the injury was assessed by a medical professional and resulted in removal from play were adapted from previous epidemiological studies in Gaelic football.^32,34,35^ The coding framework was then validated by a panel of 5 experts in either injury prevention and/or experience working with elite LGF teams and/or experience completing performance analysis in Gaelic football. These included 2 athletic therapists and 3 performance analysts. Each expert scored each criterion on the coding framework on an 11-point Likert scale (where 0 = strongly reject, and 10 = strongly accept). Criteria that achieved an average of ≥50% acceptance were included in the final coding framework (Online Supplementary Tables 1 and 2), whereas criteria that did not meet the level of acceptance were excluded. Four criteria were included to identify suspected injuries: (1) “situation in which the match was interrupted by the referee for a player in distress,” (2) “a player lay on the pitch for more than 10 seconds,” (3) “a player appeared to be in pain,” and (4) “the player received medical treatment” (Supplementary Table 1). To identify suspected concussions, 15 previously validated criteria were evaluated for their suitability in the context of Gaelic football and subsequently included in the framework (Supplementary Table 2).^45^ All video analysis and coding were conducted by a single certified athletic therapist with 5 years of clinical and pitchside experience with LGF and 1 year at the elite level. All coding was conducted on NacSport Scout (Version 9.0) and all championship and league matches are freely available on YouTube (LadiesFootballTV channel) and the TG4 Player to view. Most video footage was recorded using a single camera in an elevated position near the halfway line of the pitch. The coder’s intrareliability for recording injury criteria was examined on a sample of 14 championship and league matches from the 2021 season coded 1 week apart. Intrareliability of the coder was observed to be “almost perfect” (kappa score, 0.98), where a kappa score of >0.80 was chosen as an acceptable level of agreement.^28^ In total, 120 matches from the 2022 season were then coded. Most matches were 60-minutes in duration; however, 2 matches required extra time (additional 20 minutes per game), totaling 120.7 hours of footage. Match exposure was calculated by multiplying match hours by number of players exposed. To ensure foul play events were accurately coded, an experienced national LGF referee was recruited (17 years of experience) and coded the foul play events along with the primary coder to confirm accuracy.

### Data analysis

The incidence of suspected injuries and suspected concussions were calculated as suspected injuries/concussions per 1000 match hours alongside corresponding 95% CIs. Descriptive statistics (ie, means, medians, interquartile range, standard deviations, range, frequencies) for the other variables were calculated using IBM SPSS Statistics for Windows, Version 28.0, released 2021 (IBM Corp).

## Results

### Suspected Injury and Concussion Incidence

In total, 829 suspected injuries were observed in 120 matches (Table 1). The median number of criteria met for all suspected injuries was 4 (out of 4, interquartile range [IQR], 3-4). Suspected concussions accounted for 19.5% of all suspected injuries (*n* = 162; median criteria, 3 of 15; IQR, 2-3). The injury rate for all suspected injuries was 229.0 per 1000 player-hours (95% CI, 214.0-245.2), the rate of medical attention injury was 192.3 per 1000 player-hours (95% CI, 178.5-207.1), and the rate of suspected concussion was 44.8 per 1000 player-hours (95% CI, 38.4-52.2); Table 1). There were, on average, 6.9 ± 3.2 suspected injuries and 1.4 ± 1.1 suspected concussions per match. Of the 829 suspected injuries, 50.4% occurred in league matches, with 49.6% in championship matches.

**Table 1.** Suspected injury and concussion rates

	Competition	Number of injuries	Rate per 1000 hours (95% CI)	Rate per match (95% CI); (range)
Suspected injuries	All	829	229.0 (214.0-245.2)	6.9 (6.5-7.4); (2-17)
	Championship	411	239.0 (217.0-263.2)	7.2 (6.6-7.9); (2-16)
	League	418	220.0 (199.9-242.2)	6.6 (6.03-7.3); (2-17)
Suspected concussions	All	162	44.8 (38.4-52.2)	1.4 (1.2-1.6); (0-4)
	Championship	82	47.7 (38.4-59.2)	1.4 (1.2-1.8); (0-4)
	League	80	42.1 (33.8-52.4)	1.3 (1.0-1.6); (0-4)
Medical attention injuries	All	696	192.3 (178.5-207.1)	5.8 (5.4-6.3); (1-14)
	Championship	346	201.2 (181.1-223.5)	6.1 (5.5-6.7); (1-13)
	League	350	184.2 (165.9-204.6)	5.6 (5.0-6.2); (1-14)

### Injury Management

The majority of suspected injuries (84.0%) received onfield medical attention, while 16.0% did not receive onfield medical attention. The player was removed temporarily from play for 1.2% of suspected injuries, 6.0% were removed permanently from play, and 92.2% remained on field. Of the suspected concussions, 90.1% received onfield medical attention, whereas 9.9% did not. For suspected concussions, 3.1% were removed temporarily from play, 10.5% were removed permanently from play, and 84.6% remained on field. For 5 injuries (0.6%) and 3 suspected concussions (1.9%), it was unclear whether the player left the playing field (eg, the injury occurred at the end of the game).

### Injury Characteristics

Forwards had the highest incidence of suspected injuries (251.4 per 1000 player-hours, 95% CI, 226.9-278.6), followed by midfielders (246.6 per 1000 player-hours, 95% CI, 206.0-295.1) (Table 2). Events relating to the tackle accounted for 40.2% of all injuries, with “being tackled” (27.5%) the most frequent injury event followed by contested ball events (accounting for 20.0%). The mechanism resulting in the most injuries was player-to-player contact (68.2%), followed by contact with the ground (14.4%), whereas the mechanism could not be determined in 11.0% of cases. Just 5.1% of injuries were noncontact injuries (Table 3). Suspected injuries occurred most frequently in the fourth quarter (28.5%), followed by the third quarter (22.8%) (Table 3). Injuries to the head/neck (38.1%) and lower limb (25.8%) were the locations injured most frequently (Table 3).

**Table 2.** Suspected injury and concussion rates per playing position

		Number of suspected injuries	Suspected injury incidence rate per 1000 player-hours (95% CI)	Number of suspected concussions	Suspected concussion incidence rate per 1000 player-hours (95% CI)
Playing position	Forward	364	251.4 (226.9-278.6)	75	51.8 (41.3-65.0)
	Midfield	119	246.6 (206.0-295.1)	24	49.7 (33.3-74.2)
	Back	312	215.5 (192.9-240.8)	53	36.6 (28.0-48.)
	Goalkeeper	28	116.0 (80.1-168.1)	9	37.3 (19.4-71.7)
	Position unknown	6	-	1	-

**Table 3.** Events, mechanisms, occurrence, and locations of suspected injuries

		All suspected injuries, % (n)
Event	Being tackled	27.5 (228)
	Collision*^a^*	13.6 (113)
	Tackling	12.7 (105)
	Contested ball on the ground*^b^*	10.1 (84)
	Falling	5.9 (49)
	Contested ball in the air*^c^*	4.9 (41)
	Contested ball (other)*^d^*	4.9 (41)
	Collision with stationary defender*^e^*	4.0 (33)
	Event unknown/unclear	3.4 (28)
	Turning/changing direction/cutting	2.5 (21)
	Blocking	1.8 (15)
	Kicking	1.3 (11)
	Picking the ball up from the ground	1.3 (11)
	Diving	1.2 (10)
	Saving	1.0 (8)
	Being hit/struck*^f^*	1.0 (8)
	Jumping/landing	0.8 (7)
	Running/sprinting	0.6 (5)
	Decelerating	0.6 (5)
	Pushing/shoving	0.5 (4)
	Catching	0.2 (2)
Mechanism	Player-to-player contact	68.2 (565)
	Contact with playing surface	14.4 (119)
	Mechanism unknown/unclear	11.0 (91)
	Noncontact	5.1 (42)
	Contact with equipment (eg, ball, goalpost, etc)	1.4 (12)
Occurrence	Fourth quarter	28.5 (236)
	Third quarter	22.8 (189)
	Second quarter	19.4 (161)
	First quarter	18.9 (157)
	Additional time in second half	5.7 (47)
	Additional time in first half	3.5 (29)
	Extra time	1.2 (10)
Location of suspected injury	Head/neck	38.1 (316)
	Lower limb	25.8 (214)
	Location unknown/unclear	14.4 (119)
	Torso	11.0 (91)
	Upper limb	10.7 (89)

a An event in which ≥2 players collide with each other in a relatively short time resulting in a change to the players momentum or direction of travel.

b An event where a player(s) from either team competes for possession of a ball that is on the ground.

c An event where a player(s) from either team competes for possession of an aerial ball that is above shoulder height.

d An event where a player(s) from either team competes for possession of the ball which is neither on the ground nor above shoulder height (eg, intercepting a pass/kick).

e An event in which an attacking player collides with a deliberately stationary defender resulting in a change to the attacking player’s momentum or direction of travel.

f An event where contact is made to a player, typically with the hand, foot, or other body part, with sudden force or intent.

### Concussion Characteristics

Of the 162 suspected concussions, the incidence was greatest in forwards (51.8 per 1000 player-hours; 95% CI, 41.3-65.0) (Table 2). The tackle event contributed to the majority of suspected concussions (34.6%), followed by contested ball events (25.9%) (Table 4). Of all events, suspected concussions resulted most frequently from “collisions” (22.2%), and “being tackled” (21.6%). Player-to-player contact was the mechanism resulting in the most suspected concussions (75.3%), of which head contact with an opposing player's shoulder was most common (15.4%). Head impact with the playing surface resulted in 19.8% of suspected concussions, whereas the exact impact location could not be determined in 25.3% of cases due to obscured video footage (discussed in the limitations) (Table 4). Most suspected concussions occurred in the fourth quarter (29.0%), followed by the third quarter (25.3%) (Table 4). The most prominent criteria for suspected concussions were “grabbing/clutching of the head” (86.4%), followed by “slow to return to feet after direct/indirect head contact” (55.6%) (Table 5).

**Table 4.** Events, mechanisms, occurrence, and impact locations of suspected concussions

		All suspected concussions, % (n)
Event	Collision*^a^*	22.2 (36)
	Being tackled	21.6 (35)
	Contested ball on the ground*^b^*	13.6 (22)
	Tackling	13.0 (21)
	Contested ball in the air*^c^*	6.8 (11)
	Falling	6.2 (10)
	Contested ball (other)*^d^*	5.6 (9)
	Collision with a stationary defender*^e^*	3.7 (6)
	Picking the ball up from the ground	3.1 (5)
	Saving	1.9 (3)
	Blocking	1.2 (2)
	Pushing/shoving	0.6 (1)
	Diving	0.6 (1)
Mechanism	Player-to-player contact	75.3 (122)
	Ground contact	19.8 (32)
	Mechanism unknown/unclear	3.1 (5)
	Contact with equipment (eg, ball, goalpost, etc)	1.9 (3)
Occurrence	Fourth quarter	29.0 (47)
	Third quarter	25.3 (41)
	Second quarter	20.4 (33)
	First quarter	14.1 (24)
	Additional time in second half	6.8 (11)
	Additional time in first half	3.1 (5)
	Extra time	0.6 (1)
Impact location	Impact unknown/unclear	25.3 (41)
	Impact with playing surface	19.8 (32)
	Head-to-shoulder	15.4 (25)
	Head-to-arm	13.0 (21)
	Head-to-head	11.1 (18)
	Head-to-torso	4.9 (8)
	Head-to-hip	4.3 (7)
	Head-to-knee	3.1 (5)
	Head-to-lower leg	1.2 (2)
	Impact with equipment (eg, goalpost, ball, etc)	1.2 (2)
	Head-to-head with teammate	0.6 (1)

a An event in which ≥2 players collide with each other in a relatively short time resulting in a change to the players momentum or direction of travel.

b An event where a player(s) from either team competes for possession of a ball that is on the ground.

c An event where a player(s) from either team competes for possession of an aerial ball that is above shoulder height.

d An event where a player(s) from either team competes for possession of the ball which is neither on the ground nor above shoulder height (eg, intercepting a pass/kick).

e An event in which an attacking player collides with a deliberately stationary defender resulting in a change to the attacking player’s momentum or direction of travel.

**Table 5.** Suspected concussion criteria

Concussion signs	All suspected concussions, % (n)
Grabbing/clutching of head	86.4 (140)
Slow to return to feet after direct or indirect head contact	55.6 (90)
Motor incoordination/ataxia/staggering gait/stumbling/staggering	17.9 (29)
Lying motionless/without purposeful movement on the playing surface, for >2 seconds	8.6 (14)
Facial injury after head trauma	6.8 (11)
Clearly dazed/dinged	4.9 (8)
No protective action - floppy. Falls to the playing surface in an unprotected manner	9.7 (6)
Cervical hypotonia	3.1 (5)
Blank/vacant look	3.1 (5)
Suspected loss of consciousness	0
Tonic posturing	0
Impact seizure/convulsion	0
Definite confusion	0
Not orientated in time, place or person	0

### Foul Play

As determined by the coder and confirmed by the referee, foul play was involved in 52.3% of all suspected injuries (*n* = 434 of 829), of which, the foul was committed by the opposition to the injured player in 79.7% of foul play cases (*n* = 346), whereas the injured player was responsible for 20.3% of cases (*n* = 88). “Nontechnical fouls” (35.2%, *n* = 292) were the most common sanction by referees, where “pushing or holding an opponent” (16.3%, *n* = 135) and “charging of an opponent” (13.9%, *n* = 115) were the most common offences. The tackle (being tackled/tackling) concerned 76.7% of fouls (*n* = 333 of 434). Of the suspected injuries resulting from the tackle, “pushing or holding an opponent” (25.8%, *n* = 86), “charging” (24.0%, *n* = 80), and “bringing the hand into contact with the body of an opponent for the purpose of dispossessing her of the ball” (15.9%, *n* = 53) were the most frequent fouls. Over half of suspected concussions did not involve foul play (53.1%, *n* = 86 of 162), whereas in 40.7% of cases concerned foul play committed by the opposition (*n* = 66), and 6.2% of suspected concussions, the injured player committed the foul (*n* = 10).

## Discussion

The present study aimed to identify the rates of suspected injury and concussion and establish the specific characteristics and the onfield management of these injuries in elite LGF players. Video analysis is gaining popularity as an efficient and cost effective method of obtaining key information regarding injury incidence, mechanisms, and management of sports injuries.^1,5,11,16,44^ However, in this study, with the absence of supporting injury data, only suspected injuries can be analyzed. Using face and content validated measures of concussion and injury criteria, West et al^44^ have shown this approach be a valid and reliable method given the high level of agreement between athletic therapists/physiotherapists, sport medicine physicians, and nonmedically trained coders.

Match suspected injury rate was 229.0 per 1000 player-hours in elite women players in this study, almost 2 times higher than a similar video analysis study on collegiate women’s rugby union players (117.5 suspected injuries per 1000 player-match hours).^45^ Research not specific to LGF suggests that game speed and intensity, size of players, and physical conditioning are greater at higher levels of play^12^; however, the physical characteristics are likely different between elite women Gaelic footballers and collegiate women rugby players. Due to the physicality and full-contact nature of rugby, players are primed for impacts between players and may be less likely to remain down unless injured,^45^ whereas LGF players may not be conditioned to physical contact situations due to the noncontact rules of the game, where deliberate contact is prohibited, which could also explain the differences in suspected injury rates. The rate of suspected concussions was 44.8 per 1000 player-match hours, accounting for 1.4 suspected concussions per match, or almost one-fifth of all suspected injuries. This is higher than reported previously in collegiate LGF players (8.9%) and male and female collegiate players (10.2%, 1.2 concussions per 1000 hours).^32,42^ However, these studies recorded only time loss injuries, meaning injuries that do not result in time loss may not be included. Furthermore, underreporting of concussions is evident in Gaelic games,^30,36^ with players often continuing to play while concussed and are commonly reluctant to self-report concussions.^29^ In similar video surveillance studies, a lower suspected concussion rate was found in women rugby players (30.8 per 1000 player-hours),^45^ but a higher rate of potential concussive events (58.1 per 1000 match hours) was noted in elite male Gaelic football.^40^ Despite previous research on multiple sports observing a higher concussion incidence rate in women compared with men,^7^ the higher rate in elite male Gaelic footballers may be attributed to greater physicality and contact permitted in the male game (such as shouldering).

Championship matches had the highest incidence of suspected injuries (239 per 1000 player-hours) and suspected concussions (48 per 1000 player-hours) in the present study. Although the physical demands of elite women’s Gaelic football are comparable with those of professional women’s soccer and Australian football,^8,43^ the amateur status of Gaelic football means players often balance work or studies in tandem with their club and county sporting commitments. The Ladies’ National Football League is played typically between January and April, whereas the All-Ireland Championship is between May and August. Thus, a possible reason for the high rates of suspected injuries could be the high training loads and compacted nature of fixtures during the season. The higher injury incidence rates towards the end of the season is in agreement in other sports,^17,20,38^ proposed to be influenced by cumulative fatigue and increased competitiveness in the latter stages.^4,19^ This could also explain why the suspected concussion incidence increased as the game progressed, with the highest proportion sustained in the fourth quarter (29%). This finding is consistent with adolescent rugby players, highlighting the potential influence of fatigue on concussion risk.^21^ Furthermore, in rugby league players, fatigue has been associated with reduced tackle technique proficiency, potentially leading to unsafe tackles, which may enhance injury risk.^18^

With regard to concussion management, while 90.1% received an onfield assessment, just 1 in 10 were removed from play after a suspected concussion. Although this is higher than observed in elite male Gaelic footballers (5.0%),^41^ it is lower than in collegiate women rugby players (39.0%).^45^ The LGFA concussion management guidelines state that players exhibiting signs of a suspected concussion should be removed immediately from the field and not return to play on the same day.^23^ The LGFA rulebook further states that under instruction by the referee, a player who sustains a suspected head injury should leave the field of play temporarily for further assessment.^24,25^ However, in this study, just 3.1% of suspected concussions were removed temporarily from play. Although medical personnel are typically present at elite matches,^29^ 65.2% of amateur ladies Gaelic games players continued playing during their most recent concussion. In addition, 49.1% stated they would not report a concussion that occurred during a championship game, and 47.3% indicated they would not report a concussion if an important game was approaching.^29^ However, the most prominent suspected concussion criteria of “grabbing/clutching the head” and “slow to return to feet after direct/indirect head contact” are not specific to concussion and may be experienced for reasons outside of concussion. Therefore, the threshold for suspected concussions in this study may be considered low. However, 28.4% (*n* = 46) of all suspected concussions exhibited ≥1 of the 6 signs listed in the international consensus definitions for identifying possible concussions using video technology.^9^ Of these, just 19.6% (*n* = 9) were removed permanently from play. Although these criteria clearly have a higher diagnostic threshold, the application of these signs to female sport requires validation. The high incidence of suspected concussions observed, along with the number of players remaining in play after suspected concussions and previous risky concussion-reporting behaviors,^29,30,41^ highlights the critical need for targeted interventions not only for players but also for medical professionals, coaches, and referees at all levels.

Contact injuries in the current study (84%: contact with another player, ground contact, or equipment) were far higher than previously self-reported as mechanisms of injury in collegiate LGF players (34%) and elite male Gaelic footballers (10% to 40% contact injuries).^10,32^ Contact with another player was the most frequent mechanism for suspected injuries (68%) and suspected concussions (75%) in this study. Similar injury rates in elite women Australian footballers have been observed, where 53% of all injuries resulted from an impact/contact mechanism, of which 46% of all injuries did not result in time-loss.^14^ Regarding suspected concussions, head contact with the shoulder (15%) and head contact with the arm (13%) were the most common causes of impact. In comparison, the most common mechanism for potential concussive events in elite male Gaelic football involved contact with the hand/fist to the head (27%) or arm-to-head contact (24%),^40^ where the authors observed that 38% of potential concussive events resulted from poorly timed tackles, where the arm of the defender came into contact with the ball-carrier when trying to dispossess them of the ball. Specifically, regarding the tackle in LGF, to dispossess an opposing player of the ball, the player must knock the ball from the opposition’s hand by “flicking it with the open hand or hands;” however, if the opposition is holding the ball into her body, she cannot be legally dispossessed. Therefore, players must time the tackle when the player in possession is bouncing, soloing, kicking, or passing the ball.^24^ However, failure to accurately time the tackle can result in foul play and potential injuries. In this study, similar to previous LGF research,^32^ the tackle (40%: both tackling and being tackled) was a common mechanism of injury, with a high proportion of suspected concussions resulting from contact with another player in the tackle (35%). Over half of all suspected injuries involved foul play, with a high proportion of foul play events associated with the tackle (77%). The LGFA has produced educational content on “clean tackling” skills for players and coaches^22^; however, much like the poor uptake of injury prevention programmes,^6^ engagement with this content may currently be limited due to barriers such as accessibility, awareness, and coaching confidence. Thus, enhanced educational initiatives promoted by the LGFA that address these barriers could improve engagement and have a positive impact on injury and concussion risk. This has been demonstrated in rugby, where a nationwide injury prevention program has been associated with safer tackling techniques and a reduction in catastrophic injuries.^3^ A similar approach in LGF could ensure clean tackling education reaches its target audience effectively, leading to meaningful changes in behavior and injury risk. Contests between players for possession of the ball (20%) were also common injury mechanisms, of which over a quarter of suspected concussions resulted from contact with another player during these events. Although deliberate contact is not permitted, during 50/50 engagement for a ball, contact between players can occur. These findings further highlight that contact events need to be further addressed.

Although previous research has found lower limb injuries are predominant in LGF (67%) and account for the greatest percentage of injury claims (64%), total costs (83%), and time loss in the sport,^32,37^ we found that only 1 in 4 suspected injuries occurred to the lower limb. In contrast, the head/neck was the location injured most frequently in the present study (38%), compared with 12.7% in collegiate LGF players.^32^ A possible explanation for these differences is that much of injury data in LGF typically accounts for severe or expensive injuries sustained while playing that result in time-loss from training or matches. As a result, players who suffer “minor” injuries that may not require medical treatment are not recorded. In this study, all suspected injuries were reported; however, the severity of the suspected injuries could not be determined.

A limitation of this study is the use of a single coder, which may introduce bias and limit the diversity of interpretations. Although the coder was experienced in sports injuries and concussion protocols, the decision to use a single coder was based on resource constraints. In addition, most match footage relied on a single-camera angle, which sometimes limited visibility (eg, injuries occurring away from the camera, being obstructed by players/officials, environmental factors such as rain on the lens) and often had lower resolution. Furthermore, footage was unavailable for analysis in 5 matches due to recording errors (frozen video), resulting in a total data loss of 12 minutes and 30 seconds (19 seconds, 622 seconds, 20 seconds, 67 seconds, and 22 seconds for each respective match). In contrast, professional coverage of 21 matches provided higher resolution and multiple-angle footage, offering additional analytical opportunities, specifically when replays of the suspected injury were available. These replays often included slow-motion, close-ups, or alternative camera angles, allowing for a more detailed examination of specific events. When replays were unavailable, the analysis in professional footage was comparable with nonprofessional footage. In both professional and nonprofessional footage, suspected injuries could be identified, but specific characteristics, such as the exact mechanism or location, were sometimes obscured by camera angle limitations or obstructions. Any data that could not be confidently interpreted was classified as unclear/unknown. As a result, this methodological choice may have introduced a risk of measurement bias, potentially leading to the underreporting or misclassification of suspected injuries. Without supporting prospective injury data, true injury incidence is not confirmed, and therefore suspected injuries and concussions represent an estimate of match injury rates only. In particular, the incidence of suspected concussions might be overestimated due to the broad identification criteria—for example, “grabbing or clutching the head” may result from causes unrelated to concussion—yet underestimated as concussion symptoms may evolve over time.

## Conclusion

Given the dearth of prospective injury data in LGF, this novel method of injury surveillance provides valuable insights necessary to inform targeted injury prevention strategies for the LGFA. The incidence of suspected injuries relating to the tackle and contests for possession are higher than previously observed in LGF. Of particular concern is the onfield management of suspected concussions, specifically the high number of players who remained in play after a suspected concussive event. Overall, the findings from the study highlight the need for the development of injury prevention and concussion management strategies, as well as education for all knowledge users to promote a safer playing environment for LGF players.

## Supplemental Material

sj-docx-1-sph-10.1177_19417381251372982 – Supplemental material for A Video Analysis of Suspected Injuries and Suspected Concussions in Elite Ladies Gaelic Football Matches Supplemental material, sj-docx-1-sph-10.1177_19417381251372982 for A Video Analysis of Suspected Injuries and Suspected Concussions in Elite Ladies Gaelic Football Matches by Leigh Porter, Stephen W. West, Stephen Behan and Siobhán O’Connor in Sports Health
