# Supplementary material for: A Video Analysis of Suspected Injuries and Suspected Concussions in Elite Ladies Gaelic Football Matches
Source: Sports Health. 2025 Sep 25:19417381251372982. Online ahead of print. doi: 10.1177/19417381251372982 (PMC12463854; doi:10.1177/19417381251372982)
Supplement: sj-docx-1-sph-10.1177_19417381251372982 – Supplemental material for A Video Analysis of Suspected Injuries and Suspected Concussions in Elite Ladies Gaelic Football Matches [file sj-docx-1-sph-10.1177_19417381251372982.docx]

**Supplementary Data**

Table S1– Range of percentage agreement between individual raters for suspected injuries.

|  | **Expert 1** | **Expert 2** | **Expert 3** | **Expert 4** | **Expert 5** | **Acceptance Average** |
| --- | --- | --- | --- | --- | --- | --- |
| A situation in which the match was interrupted by the referee for someone in distress | 90 | 40 | 80 | 50 | 80 | 68% |
| A player lay on the pitch for >10s | 50 | 60 | 70 | 90 | 80 | 70% |
| Player appeared to be in pain | 60 | 90 | 70 | 100 | 50 | 74% |
| Player received medical treatment | 100 | 90 | 90 | 100 | 80 | 92% |

Table S2 – Range of percentage agreement between individual raters for suspected concussions.

|  | **Expert 1** | **Expert 2** | **Expert 3** | **Expert 4** | **Expert 5** | **Acceptance Average** |
| --- | --- | --- | --- | --- | --- | --- |
| Player unable to resume play in a meaningful capacity within 5s of direct and visible head contact | 100 | 80 | 90 | 100 | 50 | 84% |
| Loss of consciousness | 100 | 100 | 100 | 100 | 100 | 100% |
| Lying motionless | 100 | 90 | 100 | 90 | 80 | 92% |
| Motor incoordination/ataxia/staggering gait/stumbling | 90 | 100 | 90 | 100 | 80 | 92% |
| Tonic posturing | 90 | 90 | 100 | 90 | 100 | 94% |
| No protective action | 80 | 90 | 90 | 90 | 80 | 86% |
| Cervical hypotonia | 80 | 100 | 70 | 100 | 80 | 86% |
| Impact seizure/convulsion | 100 | 90 | 70 | 100 | 100 | 92% |
| Blank/vacant look | 100 | 20 | 60 | 100 | 30 | 62% |
| Clearly dazed/dinged | 90 | 100 | 80 | 100 | 70 | 88% |
| Not orientated in time/place/person | 80 | 100 | 70 | 100 | 70 | 84% |
| Slow to return to feet after direct/indirect head contact | 80 | 100 | 50 | 100 | 70 | 80% |
| Facial injury after head trauma | 80 | 80 | 20 | 100 | 50 | 66% |
| Grabbing/clutching of head | 90 | 100 | 30 | 100 | 60 | 76% |
| Suspected concussion | 100 | 10 | 100 | 100 | 0* | 62% |
| If one of the above criteria is met, a concussion is suspected. | 100 | 90 | 80 | 100 | 0* | 74% |

*No response was recorded by the rater
